# Digitizing extant bat diversity: An open-access repository of 3D μCT-scanned skulls for research and education

**DOI:** 10.1371/journal.pone.0203022

**Published:** 2018-09-18

**Authors:** Jeff J. Shi, Erin P. Westeen, Daniel L. Rabosky

**Affiliations:** 1 Museum of Zoology, University of Michigan, Ann Arbor, MI, United States of America; 2 Department of Ecology and Evolutionary Biology, University of Michigan, Ann Arbor, MI, United States of America; 3 Center for Educational Innovation, University of Minnesota, Minneapolis, MN, United States of America; Monash University, AUSTRALIA

## Abstract

Biological specimens are primary records of organismal ecology and history. As such, museum collections are invaluable repositories for testing ecological and evolutionary hypotheses across the tree of life. Digitizing and broadly sharing the phenotypic data from these collections serves to expand the traditional reach of museums, enabling widespread data sharing, collaboration, and education at an unprecedented scale. In recent years, μCT-scanning has been adopted as one way for efficiently digitizing museum specimens. Here, we describe a large repository of 3D, μCT-scanned images and surfaces of skulls from 359 extant species of bats, a highly diverse clade of modern vertebrates. This digital repository spans much of the taxonomic, biogeographic, and morphological diversity present across bats. All data have been published to the MorphoSource platform, an online database explicitly designed for the archiving of 3D morphological data. We demonstrate one potential use of this repository by testing for convergence in skull shape among one particularly diverse group of bats, the superfamily Noctilionoidea. Beyond its intrinsic utility to bat biologists, our digital specimens represent a resource for educators and for any researchers seeking to broadly test theories of trait evolution, functional ecology, and community assembly.

## Introduction

Organismal morphology is key to our conception of how species interact with one another and with their environments [1–3]. Furthermore, morphology often reflects and represents some of the clearest examples of natural selection and adaptation, both over evolutionary timescales and in response to global change. Given these considerations, physical repositories of specimens, like museums of natural history, are invaluable resources for ecologists and evolutionary biologists [4–5]. Analyzing the morphology of specimens collected for and preserved within these repositories can reveal the tempo and mode of morphological evolution [6] and species’ responses to external change [7–9], and can be a window into the scale and diversity of biological innovation [10–11]. By integrating data across these various collections, researchers can highlight broad ecological and evolutionary trends throughout branches of the tree of life and over multiple biogeographic realms.

The creation and curation of digital specimens—electronic records, visualizations, and reproductions of physical specimens—can improve accessibility and collaboration across institutions, especially when they are open-access to the research community. Some aspects of morphology that are difficult to investigate with fragile and rare physical specimens can be studied using digital specimens. For instance, some internal morphological traits cannot be measured or otherwise studied without damaging or destroying samples [12]. Digital specimens can also facilitate analysis of particularly small or cryptic aspects of morphology [11, 13–15]. Rote tasks, including measurements and character scoring, can also be automated and scripted when digital specimens are used, streamlining data collection and accelerating the pace of museums-based research.

In recent years, researchers have harnessed X-ray computed microtomography (μCT) scanning as an approach for digitally capturing and visualizing morphology in three-dimensional space. μCT scans are particularly useful for digitally imaging hard tissue in specimens, though considerable advances have been made to extend the method to soft tissue scanning [16–17]. Generalized μCT scanning methods produce high-resolution images and 3D volumes and surfaces that can be used for a variety of derived analyses, ranging from finite element analysis [18] to both traditional linear and geometric morphometrics [19–20]. Curating large repositories of μCT data is rapidly gaining traction, especially in cases where the original specimens are particularly rare and valuable [21–22].

Here, we describe a digital 3D, open-access repository of extant bat skull diversity that spans much of the phylogenetic and ecological breadth of the clade. We detail its assembly and accessibility, and discuss some of its potential uses for the general community. Bats (Mammalia: Chiroptera) are both ecologically and morphologically heterogeneous, with clear links between both axes of diversity [23–24]. The close synergy between form and function in this clade also spans multiple facets of their ecology and behavior. For instance, measurements of wing shape have been linked to dispersal ability [25], nasal and auricular geometry to echolocation broadcasting [11, 26], and jaw morphology to trophic ecology [27–28].

The shapes of bat skulls and faces, in particular, are bridges between physical performance and ecology, both externally (*e*.*g*. capturing and processing food) and internally (*e*.*g*. modulating and emitting echolocation calls). Our repository captures much of the skull shape diversity of extant bats, as it is designed to maximize sampling across both the bat phylogeny and their biogeographic distribution. Here, we describe the specimens currently available within this database, and demonstrate one potential use of the surface files with a test of shape convergence among an ecologically diverse clade of bats, the New World superfamily Noctilionoidea. Our overall goals are to provide a solid foundation for any researchers interested in bat morphology, its ecological consequences, and its evolutionary drivers.

## Materials and methods

### Specimen and collection details

We scanned adult skulls of bat specimens from the University of Michigan Museum of Zoology (UMMZ) and the American Museum of Natural History (AMNH). Sexual selection may occur in some bat species [29–30]; as such, we generally measured females, but maximized species-level taxonomic diversity whenever possible. We separated mandibles and crania for most specimens, although this was not feasible for a small number of articulated specimens. We mounted all specimens in foam to prevent movement in preparation for μCT-scanning.

For this database, we first prioritized scanning of all bat species that are present in the UMMZ collections, where scans were performed and all authors were based. As such, our sampling is heavily weighted towards those clades that are available within the UMMZ. We supplemented our database, increasing the species scanned per family, using the AMNH collections. We also prioritized specimens represented in a recent species-level phylogeny of the order [31]. We μCT-scanned 435 total skulls across the two museums: 230 skulls of specimens from the UMMZ collections and 205 skulls of specimens from the AMNH.

### μCT-scanning, image processing, and validation

All specimens were scanned and reconstructed using a μCT scanner (μCT100 Scanco Medical, Bassersdorf, Switzerland) associated with the University of Michigan School of Dentistry. We performed nearly all scans and reconstructions at a voxel size between 12 and 30 μm (with the vast majority of scans at 20 μm), with a peak kilovoltage of 70V across the X-ray tube and a current of 114 μA (S2 Table). Each scan was filtered with a 0.5 mm aluminum filter, and scanning proceeded for 750 projections with an integration time of 750 ms. Only the disproportionately large skulls of the flying fox family Pteropodidae were scanned with significantly different voxel sizes of 30–60 μm. Full scan details are available in the S2 Table.

We imported the resulting 16-bit DICOM stacks for each cranium and mandible into the program ImageJ [32], where they were cropped and edited to minimize scanning artifacts and to enhance contrast between bone and negative space. In general, editing was restricted to minimal adjustments of brightness and contrast. We then converted all images into 8-bit TIFF stacks for further processing and digital storage.

To generate 3D surfaces for all of the UMMZ specimens, we imported the specimen-specific TIFF stacks into the program Avizo 9.2.0 (FEI, Hillsboro, USA) for reconstruction and segmentation. We segmented bone from other material, such as the mounting foam, using built-in multi-thresholding and segmentation editors, and then generated three-dimensional surfaces. All thresholded and segmented surfaces were exported as PLY files for storage and broad compatibility with widely-used morphometric software.

As our goal is for digital specimens to be comparable with and used alongside physical specimens, there may be concern about how the scanning and reconstruction process may make digital measurements differ from traditional measurements. We compared linear caliper measurements taken from the original, physical specimens with electronic measurements processed in Avizo 9.2.0. These measurements are fully described by Dumont *et al*. [28], and are abbreviated as follows: MZB (maximum zygomatic breadth), TSL (total skull length), PSW (posterior skull width or mastoid breadth), SKH (skull height), PM1 (palate width at molar 1), CH (condyle height), CM1 (mandibular length from condyle to molar 1), MSW (minimum skull width), and CPH (coronoid process height).

### Testing for skull shape convergence

One potential use of this database is in phylogenetic analyses of skull shape. To demonstrate this possibility, we performed a test of skull shape convergence among New World noctilionoid bats. Noctilionoids span the full breadth of bat trophic diversity, and the clade is also where the links between skull shape and function have been best characterized [27–28]. One highly specialized trophic behavior among noctilionoids, nectarivory, arose independently within noctilionoids at least twice [33–35]. This leads to the possibility that skull shapes among nectarivores are convergent, where similar morphologies have also independently arisen across lineages.

To test this hypothesis, we selected a small subset of 30 specimens that represented both lineages of noctilionoid nectarivores, the subfamilies Lonchophyllinae and Glossophaginae [35], as well as three other major categories: insectivory, frugivory, and sanguivory. We used the trophic classifications of Rojas *et al*. [36] for these analyses, and focused on the crania for each specimen. Species included for this analysis are highlighted in the S1 Table.

We then quantified shape for these 30 cranial specimens using landmark-based geometric morphometrics and the R package *geomorph* [37]. Using the program Checkpoint (Stratovan, Davis, USA), we placed a series of 29 fixed landmarks on each cranium, as well as 15 equidistant semilandmarks along the sagittal crest. These 44 landmarks are adapted from and described in full by Santana & Lofgren [26]. All subsequent analyses were performed in *geomorph*, unless otherwise noted. We then imported these landmarks into *geomorph* and estimated any missing landmarks (*e*.*g*. on damaged specimens) by using a thin-plate spline to extrapolate from complete datasets [38]. To align these raw shape data into a common coordinate system, we used a generalized Procrustes analysis, scaled by centroid size, where semilandmarks were allowed to slide along the sagittal crest using the Procrustes distance criterion [39–40].

To test for convergence, we used the C1 statistic described by Stayton [41] and as implemented in the supplementary code of Zelditch *et al*. [42]. C1 estimates the proportion of maximum, ancestral interspecific, morphological distances that have since been minimized by putatively convergent evolution among a set of specified taxa. Significance of C1 is estimated by simulating morphological evolution under Brownian motion, estimating a simulated C1, and comparing it to the empirical estimate. We calculated C1 for the non-monophyletic trophic categories of insectivory, frugivory, and nectarivory among our 30 crania. We also tested convergence between sanguivory, the most derived of bat trophic ecologies, and each of the other three trophic guilds. For all these tests, we calculated significance by simulating shape evolution under Brownian motion 100 times, estimating C1 for each of these simulations, and calculating the proportion that exceeded our empirical estimate.

### MorphoSource storage

We archived all data on MorphoSource (http://www.morphosource.org/), an online data archive that sorts 3D datasets into individual projects for rapid dissemination and ease of sharing with collaborators and practitioners. These data were all archived under a Creative Commons license (CC-BY-NC), making them open-access to the community. Each specimen was vouchered and represented by a compressed folder of TIFF images and, for the UMMZ specimens, an associated PLY surface file.

## Results

### Repository details

**Fig 1:**
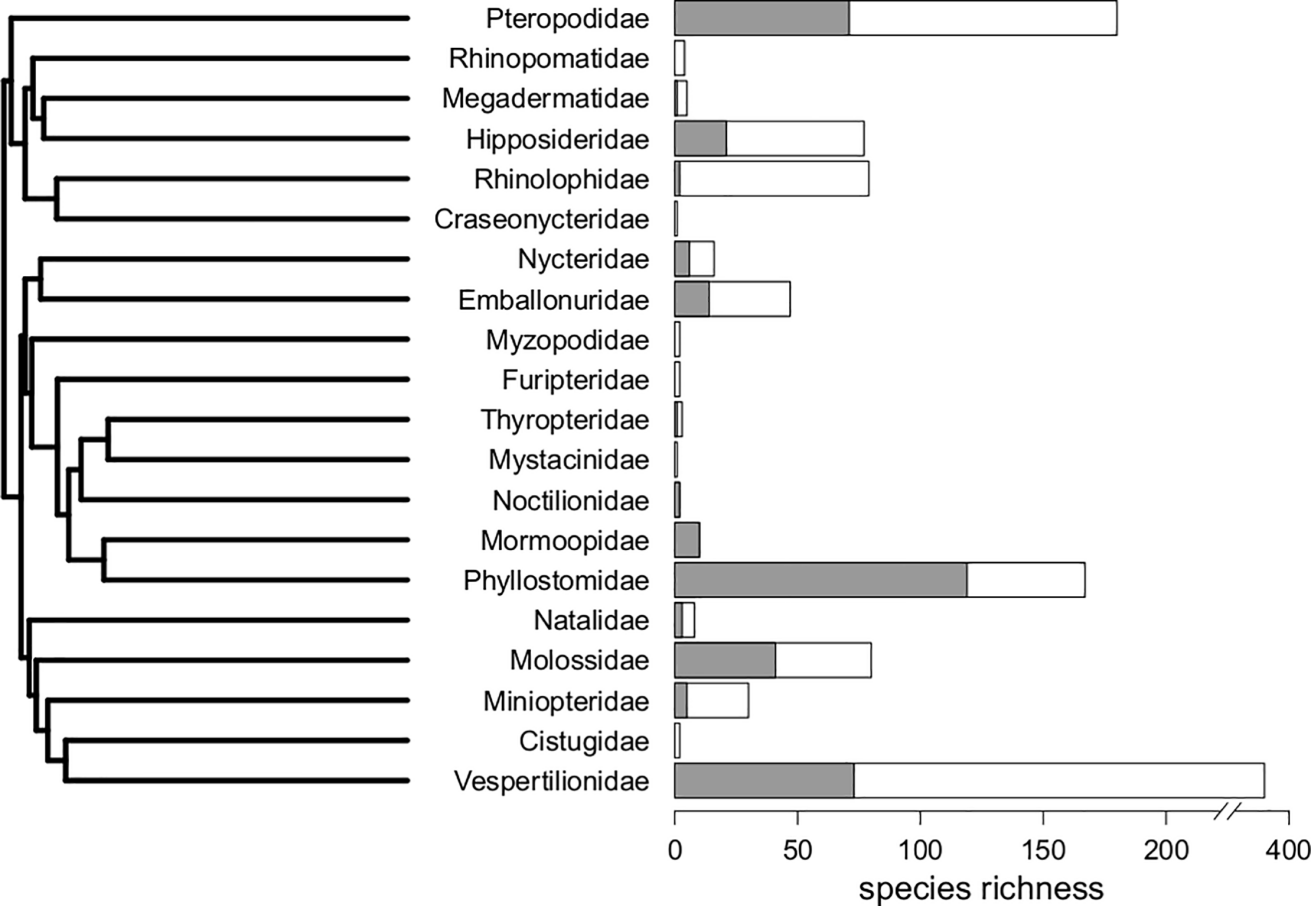
Sampling of each extant bat family within this repository. On the left, all twenty extant families of bats are displayed [24] along the phylogenetic backbone of the order [31]. On the right, estimated total richness (background, white bars) and the repository richness (filled, dark grey sections) are depicted for each family. Note that the axis for species richness is broken between 200 and 400 due to the high richness of extant vespertilionids.

Our database includes species from 14 of the 20 extant [23–24] families of bats (Fig 1). 5 of the missing families are either currently monotypic (Craseonycteridae, Mystacinidae) or monogeneric with low and particularly undersampled diversity in our source collections (Myzopodidae, Furipteridae, Rhinopomatidae). The sixth missing family, Cistugidae, is not included to avoid taxonomic misidentification: this family’s species are included in the genus *Myotis* (Family Vespertilionidae) on many databases, including iDigBio, despite recent elevation to family status [43]. Due to the potential for these species to both be mislabeled and misidentified as extremely similar *Myotis* conspecifics, we have avoided including this family until we can validate them with more specimens. Among sampled families, the Old World Rhinolophidae and Hipposideridae (superfamily Rhinolophoidea) are among the most poorly sampled in relation to their relatively high extant diversities (Fig 1).

Most species are represented by a single digital specimen, though some have multiple digital representatives in the database due to physical damage on the original specimens, or for testing intraspecific variability (see S2 Table). In total, we have 359 unique species in our repository, spanning roughly 30% of extant diversity [24, 31] (Fig 1). We note that bat taxonomy, as is true of many clades, has constantly evolved over the course of specimen collection at both institutions. As such, species names for some specimens are not always consistent across databases. The hierarchy of genera, species, and subspecies are notably in flux for many bat taxa we include here. Our count of 359 species reflects taxonomy as defined by our our species-level molecular phylogeny [31]. However, if we use iDigBio taxonomy, which is automatically associated with MorphoSource, we count 344 species, as many putative species are considered subspecies according to this taxonomy. We make note of these discrepancies and changes to taxonomy in the S2 Table.

**Fig 2:**
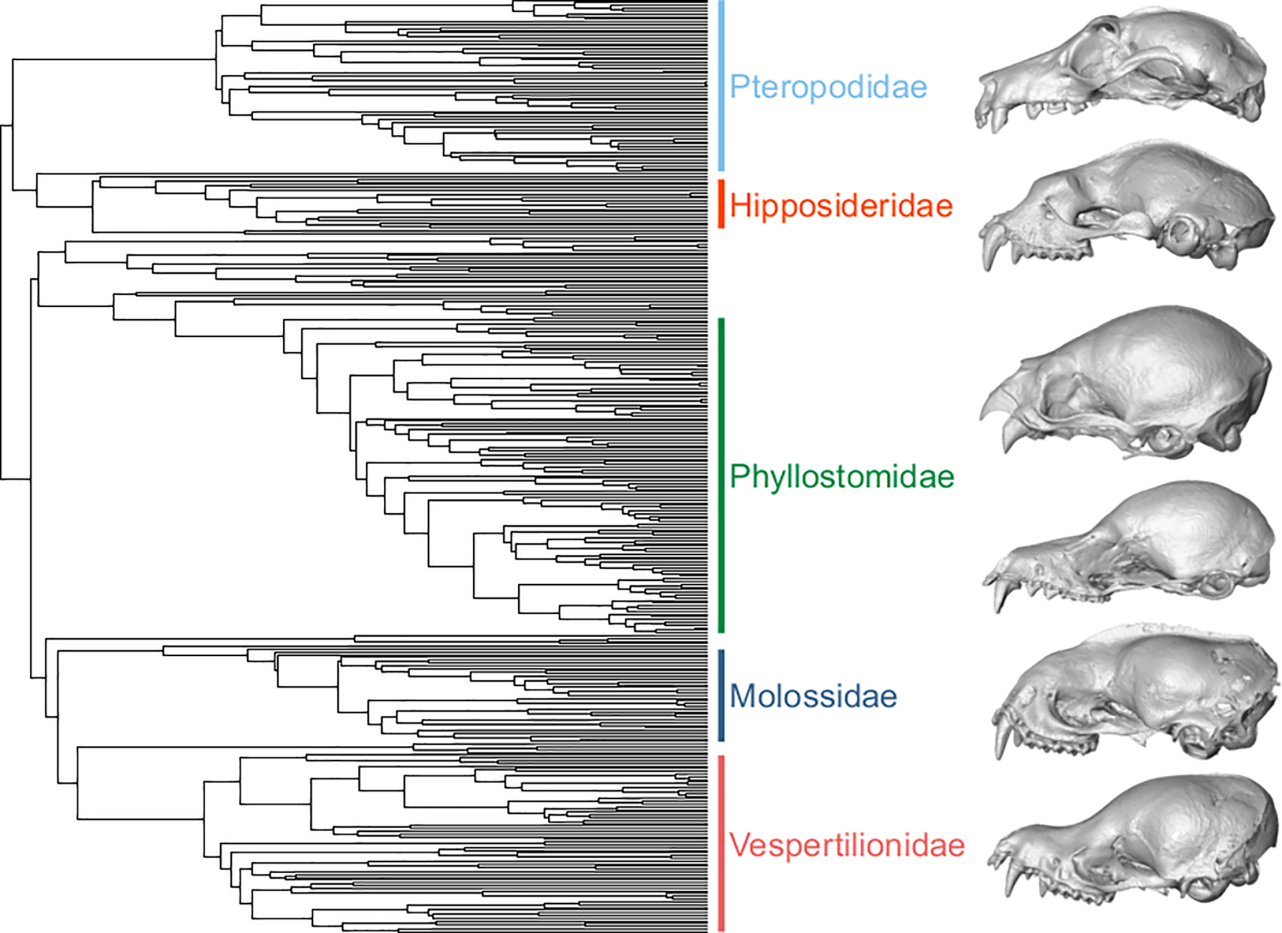
Phylogeny of species included in this repository. The phylogeny of bats included in this repository, with the most well-sampled families labeled. We include some examples of cranial surfaces from this repository for these labeled clades, to showcase the breadth of morphological disparity contained in this database, and its link to trophic diversity. Skulls are not to scale, as these species are of considerably different sizes. Species are listed as follows from top to bottom, with their associated specimen identification information from the University of Michigan’s Museum of Zoology (UMMZ) or the American Museum of Natural History (AMNH): *Pteropus hypomelanus* (UMMZ 130417; frugivore), *Hipposideros abae* (AMNH 49120; insectivore), *Desmodus rotundus* (UMMZ 116246; sanguivore), *Glossophaga leachii* (AMNH 185970; nectarivore), *Molossus molossus* (AMNH 78895; insectivore), *Myotis macrotarsus* (UMMZ 160308; insectivore).

These species span all biogeographic realms as defined by Olson *et al*. [44], and also cover all major trophic classifications of extant bats, including insectivory, nectarivory, frugivory, sanguivory, carnivory, and piscivory [23, 45]. Skulls of bats with different trophic behavior are notably distinct across the phylogeny (Fig 2).

### Scanning output details and measurement comparisons

**Fig 3:**
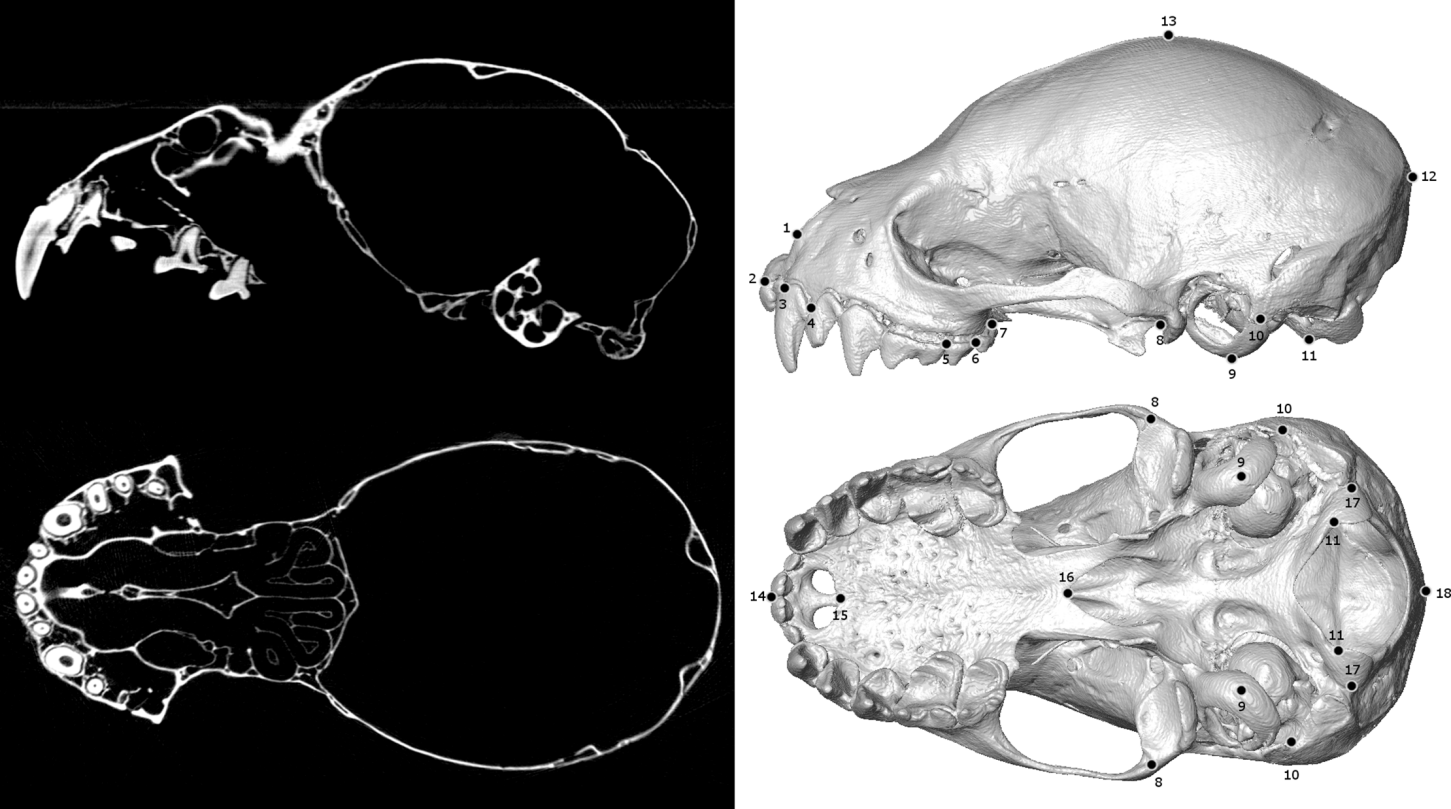
Examples of TIFF slices, a representative PLY surface, and a landmarked sample. For a specimen of *Artibeus aztecus* (UMMZ 110526), we have included two examples of individual TIFF files (left), and two analogous views of the PLY surface file (right) with labels for the fixed landmarking scheme of Santana & Lofgren [26] that we used to test for convergence in our example analysis. Full details on these landmarks can be found in that original publication. This species is generally considered a frugivore, with opportunistic consumption of insects [36].

File size and image count vary depending on length of an individual cranium or mandible and voxel size, but most surfaces are approximately 500MB with between 400–1000 individual images in their associated TIFF stack. We illustrate one example of individual TIFF files (“slices” of the overall scan) and their associated PLY surface, with our landmarking scheme used to test convergence, in Fig 3. For this database, we chose to include surfaces as PLY files, as they can easily be imported into software designed for linear morphometrics or geometric morphometrics (*e*.*g*. the package *geomorph* used in this study). Researchers can create their own surface files in other formats from the original TIFF stack, especially if they desire higher resolution or surface fidelity than is feasible for bulk online storage.

**Fig 4:**
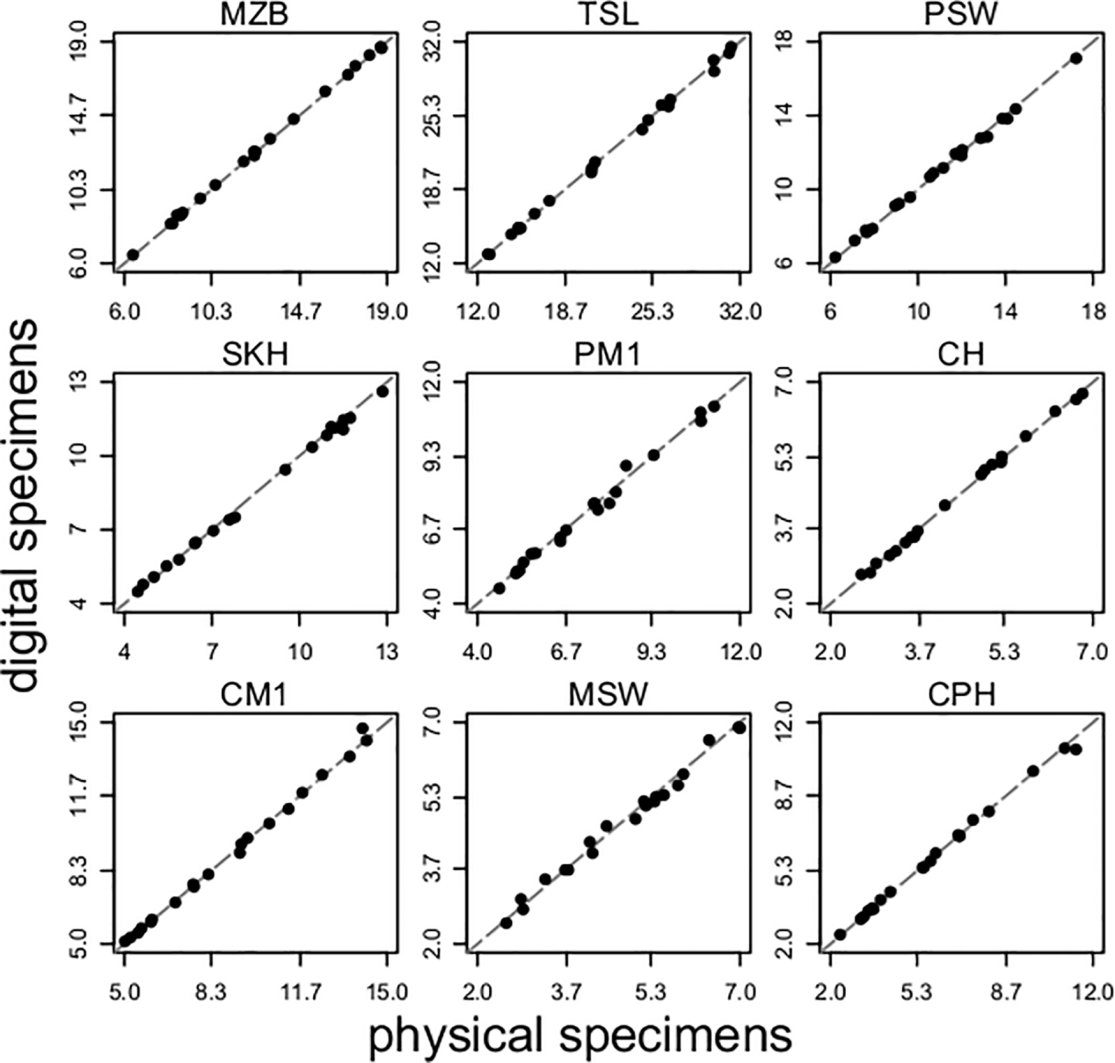
Relationships between measurements taken from digital and physical specimens. For each of 9 linear measurements, we present the relationship between measurements taken using calipers on physical specimens versus measurements taken on the surface of digital specimens, across 20 different bat species. A dashed 1:1 line is included for each measurement. Differences between the two methods are minimal and appear random with respect to species, with all *R*^*2*^ values rounding to 0.99 and 1.00 depending on precision (all *p* < 0.05).

We estimate how a basic morphometric analysis may differ based on usage of digital or physical specimens by comparing a set of nine previously-described linear measurements [28] taken using caliper measurements on physical specimens with those taken in digital space using Avizo 9.2.0. Across 20 different specimens, all of different species, we find physical and digital measurements differ by less than 2% for all measurements, on average (Fig 4, S3 Table).

### Convergence in skull shape

Of the largest three trophic categories of insectivory (12 species), frugivory (6 species), and nectarivory (9 species), only nectarivores are significantly convergent based on the C1 statistic (Table 1), despite being non-monophyletic and belonging to two separate subfamilies [35]. Interestingly, grouping nectarivores with the sanguivorous vampire bats also results in a significant C1 statistic, despite qualitative differences in skull shape (Fig 2). However, this is not true of other groupings with sanguivores. It is unclear whether this interguild skull shape convergence reflects a possible ancestral state for vampire bats, or similar biomechanical requirements for predominantly liquid diets. We hope that this brief example illustrates how this rich dataset of complex shape data can be used to illuminate issues in both ecology and evolution, as well as highlight new avenues to pursue.

**Table 1:** C1 statistic for skull shape among major trophic categories of noctilionoid bats.

guild(s)	C1 statistic	p-value
**insectivory**	0.015	1
**frugivory**	0.029	0.7
**nectarivory**	0.207	< 0.001
**insectivory + sanguivory**	0.016	1
**frugivory + sanguivory**	0.1	0.08
**nectarivory + sanguivory**	0.129	< 0.001

In Table 1, we list the largest trophic guilds of noctilionoids, as classified by Rojas *et al*. [36], and the C1 statistic and significance for testing skull shape convergence. Among the three largest guilds (excluding vampires), only nectarivores are significantly convergent based on this statistic. According to this statistic, nectarivores are also significantly convergent with vampire bats (3 species).

## Discussion

We created and shared a digital repository of 3D μCT morphological data for 359 species of extant bats. The data are publicly and freely available through the MorphoSource portal (Project #386), for immediate use or collaboration by any researchers. Several limitations of our repository should be acknowledged. While we only include skeletal data at this time, diffusible iodine-based contrast-enhanced μCT (diceCT) scans have highlighted the functional diversity of soft tissue like muscle and cartilage in bats [46], and can be integrated into this repository. Higher-resolution scanning, in general, in conjunction with soft tissue data can illuminate aspects of morphology that are not as clear at our current resolution, such as the turbinates [14]. We also recognize that our database is disproportionately biased towards New World bats, due to their representation within the UMMZ collections, where we processed all of our scans. Old World rhinolophoids and the cosmopolitan, insectivorous vespertilionoids, which together comprise the vast majority of unsampled species (Fig 1), are a natural target for future sampling and addition to this database by researchers from other institutions around the world.

We emphasize that digital databases should not be viewed as permanent replacement for primary, vouchered materials. Museum collections, despite often being critically underfunded and underappreciated, play important roles in society, policy, and education [47–49]. Digital specimens will not replace museums and their collections. Instead, we believe that digital repositories can actually highlight the extent to which museums are critical to modern research, and thus should be viewed as natural extensions of an institution's mission of promoting specimen collection. For example, digital specimens can be used to pilot initial studies, before undertaking a more expansive project housed within the physical collections or with long-term loans. Other researchers have started referring to digital specimens as “cybertypes,” analogous to the holotypes of more traditional museums-based research [50].

Accessibility to and usage of museum collections will also be improved by further digitization of specimens. Groups and individuals at institutions without affiliated museums, or who lack resources to travel can also benefit from access to a freely available database like ours. The carbon footprint of specimens-based research can even be reduced across the board by minimizing the travel and transport costs associated with physical specimens. Digital specimens can streamline and improve many typical protocols and studies of museum specimens. CT software like Avizo can accommodate specimens with sizes and shapes that preclude accurate or precise caliper measurements. For instance, users can easily incorporate curvature and volumetric calculations alongside more standard point-to-point measurements or maximum distances along anatomical axes. Volumetric data could be used for comparative studies on the evolution of internal cavities related to sensory behavior, where physical specimens may be challenging to use without damaging or destroying them.

Digital specimens also have clear utility as teaching tools that can promote discussions about a variety of ecological and evolutionary processes to students of all levels. With freely available specimens, instructors can design curricula around specimens that are fragile, rare, or otherwise unavailable for use in physical form. Finally, we emphasize that all repositories, including ours, can only be improved through collaboration with other researchers and institutions. Unlike with genetic data and GenBank, high-resolution morphological data are not currently widely archived and shared online. The sheer quantity of data produced by these analyses, interoperability, and accessibility to researchers have made this a particularly challenging endeavor [51]. However, just as the availability of genetic data through GenBank catalyzed rapid innovation in phylogenetic research, we believe that widespread community adoption of MorphoSource’s open-access model for sharing digital specimens will lead to similar advances in morphological research [52]. Our hope is that by building this digital library of phenotypes, we will facilitate increased cooperation among researchers and collections around the world, promote shared standards for similar databases, and expand the scope of possible research on form and function across the tree of life.

## Supporting information

S1 Table Species used for testing cranial shape convergence. The 30 species used to test for cranial shape convergence among the major trophic guilds of noctilionoid bats, and their families, and subfamilies, as described by Rojas *et al*. (2018). Metadata for these species is included in the full S2 Table of repository details.
(XLSX)

S2 Table Repository specimen details. A full table describing the 435 specimens contained in this repository at the time of manuscript submission, taxonomy information, sex, and associated identifiers from their parent institutions. We also note where GenBank taxonomy (our primary classification system) diverges from that of iDigBio, which is automatically associated with all MorphoSource data. Finally, we note the cases where scans were not performed at our standard 20 μm.
(XLSX)

S3 Table Percentage difference between physical and digital measurements. 20 UMMZ specimens of bats, their taxonomic and museum identification information, and differences in 9 linear measurements taken from physical and digital specimens. The measurements are described in full by Dumont *et al*. (2012), and are abbreviated MZB (maximum zygomatic breadth), TSL (total skull length), PSW (posterior skull width or mastoid breadth), SKH (skull height), PM1 (palate width at molar 1), CH (condyle height), CM1 (mandibular length from condyle to molar 1), MSW (minimum skull width), and CPH (coronoid process height). For each specimen and measurement, we calculated the percentage that digital measurements in Avizo differ from physical measurements taken using calipers. These data are also displayed in Fig 4.
(XLSX)

S1 Video *Acerodon jubatus* cranium volumetric data. A short video clip of a *Pteropus vampyrus* (an obligate frugivore) cranium rotating in 3D space. Colors and translucence represent relative density of bone material, with warmer colors and more opaque regions being more dense.
(MPG)

S2 Video *Desmodus rotundus* cranium volumetric data. A short video clip of a *Desmodus rotundus* (the common vampire bat) cranium rotating in 3D space. Colors and translucence represent relative density of bone material, with warmer colors and more opaque regions being more dense.
(MPG)
